# Computerization and Digital Workflow in Medicine: Focus on Digital Dentistry

**DOI:** 10.3390/ma13092172

**Published:** 2020-05-08

**Authors:** Marco Tallarico

**Affiliations:** Department of Periodontology and Implantology, University of Sassari, 07021 Sassari, Italy; me@studiomarcotallarico.it

**Keywords:** digital medicine, digital dentistry, mathematical models, digital workflow

## Abstract

Continuously evolving technologies make dentistry one of the most advanced sectors in the field of medicine. The digital improvements in recent years have brought many advantages to clinicians and patients, including reduced working times, lower costs and increased efficiency of performance. Some of the most important digital technologies introduced in the dental filed are cone beam computer tomography (CBCT) scan, Computer Aided Design/Computer Aided Manufacturing (CAD-CAM) systems, and intraoral scanners. All of these allow faster and more accurate rehabilitations, with the opportunity of pre-simulation of the final treatment. The evolution of computer science has brought significant advantages in the medical and dental fields, making the diagnosis and execution of even complex treatments, such as implantology and bone reconstruction, possible. The digital world is trying to supplant the traditional analog workflow, and over time, with the further advance of technologies, it should tend to be the treatment of choice of our patients.

Digital Health, is an innovative approach to medicine. The broad scope of digital health includes the computerization of healthcare processes, digitization of medical documents, computerization of medical records, creation of electronic files, and electronic prescriptions [1,2]. Examples of digital health products include software as a medical device (SaMD), mobile medical applications (MMA), software in a medical device (SiMD), and general wellness products. All of these technologies may support clinicians and patients in data collection and lifesaving applications, but also diagnosis and treatment plan understanding and execution.

The digitalization of medical records allows the clinician to share and update the patients’ informations in real time, and archive the documents more easily, with full respect for the general data protection and privacy requirements, also using cloud technologies. Instead, analog medical records are usually stored in "physical" environments such as archives or warehouses, which everyone can access. Digital documents contain sensitive data, hence it is of importance that the digitized medical records can be examined only by operators, based on an approved workflow, which was previously established. There may be many attempts to hack digital information (data breach), so it is necessary to protect them with certified procedures, starting from the software houses and ranging to the person authorized to treat them.

An example of lifesaving technological solution is geolocation, which can be used to quickly locate an emergency situation that requires the intervention of rescuers. Some emergency interventions would not be possible without the availability of satellite tracking systems and the app map-based running on smartphones [3,4]. Other eHealth technologies that could be useful to everyone and prove to be life-saving are the capabilities of emergency care using the application of smartphones, such as the SOS Emergency® (Apple iOS®) and the ELS® (Android®) [5].

The digitization of the medical profession, in all sectors, advances inexorably and offers advantages that combine analog workflows with new technologies. Digital technologies can support clinicians to make accurate diagnostic-based decisions, as well as supporting patients to make better-informed consent about their own treatment. Moreover, new options for facilitating the prevention or early diagnosis of life-threatening diseases, and management of chronic conditions outside of traditional care settings, can be provided using an innovative digital health approach. In the dental field, there are several specialities that have undergone evident changes in all the steps of the protocols and materials, including but not limiting to orthodontics, implantology, prostheses, and all the dental laboratory procedures. This allows for accurate prosthetically/functional-driven planning, proper aesthetic/functional pre-evaluation of the proposed therapy, computer-assisted execution of the treatment, as well as continuous follow-up of the patients. The main revolution was the Computer-Aided Design/Computer-Aided Manufacturing (CAD-CAM) technology, initially proposed to increase the performance of the restorations, reduce the costs and the fabrication times, and improve patient satisfaction/aesthetics. The CAD-CAM technologies make both the project and the execution faster, consisting of a three-dimensional design (planning/project) of the virtual treatment through the use of a computer, that than results in computer-assisted production of the same by the use of milling or printing machines. This technology offers several advantages, such as the accuracy of a computer-based planning/project, the rapidity of digital impression, the quality of digital-made products and its reproducibility at any time. Another very important advantage is a three-dimensional pre-visualization (simulation) that allows the final object/execution to be shown on the screen, so that the clinician can evaluate it from every viewpoint, increasing diagnostic capabilities and the accuracy of the treatments. All of this also allows for rapid communication between clinicians, other collaborators, and patients [6,7].

Some of the last considerable innovations in digital medicine are the digital impressions, optically detected by intraoral scanners (IOS), the introduction of the Cone-Beam Computerized Tomography (CBCT) scan, and the combination of both, thanks to which diagnosis and planning are faster, predictable and safe. There are various types of IOS and CBCT on the market that depend on the acquisition technology, the possibility of processing the files and the type of file generated.

Intraoral scanners use structured light scanning, which consists of projecting a grid onto the surface of the tooth, where a series of high-resolution cameras reads its distortion. The detected distortion is processed by a microprocessor which transforms this data into a dimensionally perfect object, visible directly in the acquisition software. The intraoral scanner is the perfect completion for all CAD-CAM productions; the main benefit is the possibility of immediately checking the level of accuracy of the impression with the patient still in the chair. Another great advantage is the possibility of analyzing the occlusal relationships between arches, so as to define whether the occlusal distance is suitable for the creation of CAD-CAM restorations with specific materials. Last but not least is the possibility to send impressions by email, avoiding a waste of time for delivery.

Some of the major applications in the dental field are:

1) Night guards;
2) Dental aligners;
3) Fixed dental prosthesis;
4) Guide for dental surgery.

Its increasing use is also linked to implantology, in which scan bodies are used instead of conventional transfers. Implants are then repositioned in this precise position due to CAD software that matches the specific geometric shape of the scan body, with its dedicate library, allowing the design of individual abutments, frameworks and crowns. It has been demonstrate that the system is precise and accurate, since it does not suffer from distortions due to the traditional impression of the transfers. Moreover, the greater precision in the digital impression techniques has allowed its use, in combination with Digital Imaging and COmmunications in Medicine (DICOM) data derived from CBCT, for even more accurate diagnosis and virtual implant planning, including the production of surgical guides [8,9]. For the latter, through dedicated software, the clinician can match common reference points in the STereo Lithography interface format (.Stl) files derived from intraoral acquisition and the DICOM files from CBCT scan. Finally, virtual implant planning can be done according to a prosthetic setup-up, and surgical template can be produced using a 3D printer [10].

In orthodontics, the introduction of intraoral and facial scanners, 3D printing machines, and, before that, digital radiology, including a CBTC scan, have improved both the diagnosis and execution of the orthodontic treatment. Digital studio models offer a viable alternative to traditional plaster models. Their advantages in orthodontic diagnosis and treatment planning include the easier and quicker transfer of electronic data, immediate consultation, and reduced space for storage [11]. Digital impressions/models could be analyzed by means of dedicate orthodontic analysis software able to analyze teeth, arc shape, the amount of crowding or spacing, type of malocclusion, etc. Basic and advanced measurements, including but not limited to overjet, overbite, tooth size, arch length, transverse distances and Bolton discrepancy, could be measured. All of this allows to simulate and pre-visualize the result of the orthodontic treatment. Moreover, the digital model can be finally printed with prototyping technology for diagnosis or treatment purposes. The most common application of 3D printers in orthodontics is the manufacture of aligners. Other applications are the fabrication of guides for the indirect bandaging of brackets, the production of retainers and appliances for night apneas [12,13,14,15].

Aesthetics is another application that benefits from intraoral scanners and digital workflow, consisting of smile design and simulation, prototype mock-ups, and veneer manufacturing. This is possible by the acquisition of digital impressions, a series of photos of the face and the patient’s smile, and through a smile design software that allows to shape the entire appearance of the smile area. In this way, clinicians have the opportunity to discuss with the patient and to decide with him/her the aesthetics of the restoration before the treatment starts [16,17,18]. This point is crucial to understanding patient’s expectations.

Even complete or partial prostheses, on both natural teeth and implants, could be made through a fully digital workflow, that allows clinicians and dental technicians to fabricate the restoration in all of its aspects, potentially reducing production times, and therefore the overall waiting times for both clinicians and patients, and, not least, the costs [19,20,21,22].

Every treatment starts with the acquisition of the dental impressions which can be quickly detected using an intraoral scanner (or digitalizing conventional plaster models using extraoral scanners). The digital models are processed with dedicated CAD software. Through this digital workflow, there is no need to physically deliver the impressions to the dental technicians, and this also allows the process to be safer from a biological point of view [23,24,25]. Dental technicians can then work in a fully digital way, or the digital models could be sent for printing. Once the resin model has been printed with a dedicated 3D printer machine, it is necessary to complete the hardening of the material through a post-curing process that takes place by exposing the 3D-printed object to a UV-light-curing unit for a time that varies depending on the size of the object [26,27,28,29].

Even if working in a fully digital work-flow, simple and complex treatments could be performed. As in the clinic, patient’s aesthetic and virtual jaw’s movements can be simulated before the treatment, allowing both clinicians and dental technicians to better evaluate the proposed treatment plan, and to evaluate in advance the overall cost and time required for the treatment [30,31,32,33,34,35].

In conclusion, it is true that these technologies will need to be further improved, and strong scientific evidence presented, before replacing the analog procedures. There are different aspects that need to be evaluated, therefore, both by the clinicians and patients, and in this way they could decide how to face the necessary treatments and feel more relaxed. 

## References

[1] Hasenfuß G., Vogelmeier C.F. (2019). Digital medicine. Internist.

[2] Coravos A., Goldsack J.C., Karlin D.R., Nebeker C., Perakslis E., Zimmerman N., Erb M.K. (2019). Digital Medicine: A Primer on Measurement. Digit. Biomark..

[3] Topol E.J. (2019). A decade of digital medicine innovation. Sci. Transl. Med..

[4] Adams T., Connor M., Whittaker R. (2019). Protecting our digital medicine infrastructure. NPJ Digit. Med..

[5] Fogel A.L., Kvedar J.C. (2018). Artificial intelligence powers digital medicine. NPJ Digit. Med..

[6] Warraich H.J., Califf R.M., Krumholz H.M. (2018). The digital transformation of medicine can revitalize the patient-clinician relationship. NPJ Digit. Med..

[7] Steinhubl S.R., Topol E.J. (2018). Digital medicine, on its way to being just plain medicine. NPJ Digit. Med..

[8] Spagnuolo G., Ametrano G., D’Antò V., Formisano A., Simeone M., Riccitiello F., Amato M., Rengo S. (2012). Microcomputed tomography analysis of mesiobuccal orifices and major apical foramen in first maxillary molars. Open Dent. J..

[9] Tallarico M., Martinolli M., Kim Y.-J., Cocchi F., Meloni S.M., Alushi A., Xhanari E. (2019). Accuracy of Computer-Assisted Template-Based Implant Placement Using Two Different Surgical Templates Designed with or without Metallic Sleeves: A Randomized Controlled Trial. Dent. J..

[10] Tallarico M., Kim Y.-J., Cocchi F., Martinolli M., Meloni S.M. (2018). Accuracy of newly developed sleeve-designed templates for insertion of dental implants: A prospective multicenters clinical trial. Clin. Implant Dent. Relat. Res..

[11] Giudice G., Lipari F., Lizio A., Cervino G., Cicciù M. (2012). Tooth fragment reattachment technique on a pluri traumatized tooth. J. Conserv. Dent..

[12] Cervino G., Montanari M., Santonocito D., Nicita F., Baldari R., De Angelis C., Storni G., Fiorillo L. (2019). Comparison of Two Low-Profile Prosthetic Retention System Interfaces: Preliminary Data of an In Vitro Study. Prosthesis.

[13] Fiorillo L., D’Amico C., Turkina A.Y., Nicita F., Amoroso G., Risitano G. (2020). Endo and Exoskeleton: New Technologies on Composite Materials. Prosthesis.

[14] Scrascia R., Fiorillo L., Gaita V., Secondo L., Nicita F., Cervino G. (2020). Implant-Supported Prosthesis for Edentulous Patient Rehabilitation. From Temporary Prosthesis to Definitive with a New Protocol: A Single Case Report. Prosthesis.

[15] Ortensi L., Vitali T., Bonfiglioli R., Grande F. (2019). New Tricks in the Preparation Design for Prosthetic Ceramic Laminate Veeners. Prosthesis.

[16] De Stefano R., Bruno A., Muscatello M.R., Cedro C., Cervino G., Fiorillo L. (2019). Fear and anxiety managing methods during dental treatments: A systematic review of recent data. Minerva Stomatol..

[17] De Stefano R. (2019). Psychological Factors in Dental Patient Care: Odontophobia. Medicina.

[18] Fiorillo L., Laino L., De Stefano R., D’Amico C., Bocchieri S., Amoroso G., Isola G., Cervino G. (2019). Dental Whitening Gels: Strengths and Weaknesses of an Increasingly Used Method. Gels.

[19] Cervino G., Fiorillo L., Arzukanyan A.V., Spagnuolo G., Cicciu M. (2019). Dental Restorative Digital Workflow: Digital Smile Design from Aesthetic to Function. Dent. J..

[20] Cervino G., Fiorillo L., Arzukanyan A., Spagnuolo G., Campagna P., Cicciù M. (2020). Application of Bioengineering Devices for the Stress Evaluation in Dentistry: The Last 10 Years Fem Parametric Analysis of Outcomes and Current Trends. Minerva Stomatol..

[21] Lavorgna L., Cervino G., Fiorillo L., Di Leo G., Troiano G., Ortensi M., Galantucci L., Cicciù M. (2019). Reliability of a virtual prosthodontic project realized through a 2d and 3d photographic acquisition: An experimental study on the accuracy of different digital systems. Int. J. Environ. Res. Public Health.

[22] Cicciù M., Fiorillo L., D’Amico C., Gambino D., Amantia E.M., Laino L., Crimi S., Campagna P., Bianchi A., Herford A.S. (2020). 3D Digital Impression Systems Compared with Traditional Techniques in Dentistry: A Recent Data Systematic Review. Materials.

[23] Fiorillo L. (2019). Chlorhexidine Gel Use in the Oral District: A Systematic Review. Gels.

[24] Cicciu M., Fiorillo L., Herford A.S., Crimi S., Bianchi A., D’Amico C., Laino L., Cervino G. (2019). Bioactive Titanium Surfaces: Interactions of Eukaryotic and Prokaryotic Cells of Nano Devices Applied to Dental Practice. Biomedicines.

[25] Laino L., Cicciù M., Fiorillo L., Crimi S., Bianchi A., Amoroso G., Monte I.P., Herford A.S., Cervino G. (2019). Surgical Risk on Patients with Coagulopathies: Guidelines on Hemophiliac Patients for Oro-Maxillofacial Surgery. Int. J. Environ. Res. Public Health.

[26] Lo Giudice G., Lo Giudice R., Matarese G., Isola G., Cicciù M., Terranova A., Palaia G., Romeo U. (2015). Evaluation of magnification systems in restorative dentistry. An in-vitro study. Dent. Cadmos.

[27] Cicciù M., Bramanti E., Cecchetti F., Scappaticci L., Guglielmino E., Risitano G. (2014). FEM and Von Mises analyses of different dental implant shapes for masticatory loading distribution. ORAL Implantol..

[28] Cervino G., Fiorillo L., Spagnuolo G., Bramanti E., Laino L., Lauritano F., Cicciù M. (2017). Interface between MTA and dental bonding agents: Scanning electron microscope evaluation. J. Int. Soc. Prev. Community Dent..

[29] Cicciù M., Risitano G., Lo Giudice G., Bramanti E. (2012). Periodontal health and caries prevalence evaluation in patients affected by Parkinson’s disease. Parkinson’s Dis..

[30] Cicciù M. (2019). Prosthesis: New Technological Opportunities and Innovative Biomedical Devices. Prosthesis.

[31] Hodson R. (2018). Digital revolution. Nature.

[32] Makin S. (2018). Searching for digital technology’s effects on well-being. Nature.

[33] Meloni S.M., Lumbau A., Baldoni E., Pisano M., Spano G., Massarelli O., Tallarico M. (2020). Platform switching versus regular platform single implants: 5-year post-loading results from a randomised controlled trial. Int. J. Oral Implantol..

[34] Tallarico M., Scrascia R., Annucci M., Meloni S.M., Lumbau A.I., Koshovari A., Xhanari E., Martinolli M. (2020). Errors in Implant Positioning Due to Lack of Planning: A Clinical Case Report of New Prosthetic Materials and Solutions. Materials.

[35] Meloni S.M., Spano G., Ceruso F.M., Gargari M., Lumbau A., Baldoni E., Massarelli O., Pisano M., Tallarico M. (2020). Upper jaw implant restoration on six implants with flapless guided template surgery and immediate loading: 5 years results of a prospective case series. ORAL Implantol..

